# A simple visual algorithm incorporating the components of a routine CMR study improves the determination of infarct age compared with T2-CMR alone

**DOI:** 10.1186/1532-429X-15-S1-O73

**Published:** 2013-01-30

**Authors:** Martijn W Smulders, Sebastiaan C Bekkers, Han W Kim, Lowie M Van Assche, Michele Parker, Raymond J Kim

**Affiliations:** 1 Cardiology, Maastricht University Medical Center, Maastricht, Netherlands; 2 Duke Cardiovascular Magnetic Resonance Center, Duke University Medical Center, Durham, NC, USA

## Background

T2-weighted CMR is sensitive but perhaps not specific for detecting acute MI, because T2-hyperintensity can persist for months [1]. Cine and delayed-enhancement (DE)-CMR may help in determining infarct age, since increased end-diastolic-wall-thickness (EDWT) and microvascular obstruction (MO) are frequently found in <1-month-old (acute) MI but not in 1-6-months-old (intermediate-aged) MI [1]. Given that EDWT and MO potentially resolve before T2-hyperintensity, we hypothesized that a simple, visual algorithm incorporating these components with T2-CMR could improve the determination of infarct age.

## Methods

221 CMR studies were performed at various time points post-MI in 117 STEMI patients enrolled prospectively and consecutively at two centers. True MI age was known given the STEMI date. Images were scored blinded to identity and clinical information. Pre-specified markers of acute MI were: hyperintensity on T2-CMR, MO on DE-CMR, and increased-EDWT (>150% of remote) on cine-CMR. Our algorithm incorporating multiple CMR components was based on: 1) EDWT and MO resolve before T2-hyperintensity, 2) since T2-hyperintensity eventually disappears, T2-size becomes smaller than infarct size over time.

## Results

Mean age was 58±11 years. Table 1 shows the diagnostic performance of CMR for discriminating <1 from ≥1-month-old-MI as (a) individual components, (b) basic combinations, and (c) using new algorithm. T2-CMR-alone was sensitive (88%) but not specific (66%) for <1-month-old-MI resulting in only moderate accuracy (77%). Using a later cutpoint for ‘acute' MI (2-months or 3-months) did not improve accuracy since sensitivity decreased with increasing specificity. MO and increased-EDWT were very specific but not sensitive for acute MI. The basic combination of MO-or-increased-EDWT improved sensitivity (73%) while retaining specificity (97%). Basic algebraic combinations including T2-CMR did not improve overall accuracy since ‘OR' function led to low specificity while ‘AND' function led to low sensitivity. The new algorithm resulted in high sensitivity (92%) and specificity (90%). Accuracy (91%) was improved compared with T2-CMR alone (p<0.001) and compared with basic algebraic combinations involving T2-CMR (p<0.05).

**Table 1:** Diagnostic performance of CMR for discriminating acute (<1-month-old) MI

		Sensitivity (%)	Specificity (%)	Accuracy (%)
**Individual CMR components**	T2 hyperintensity	88	66	77
	
	Increased-EDWT	42	99	70
	
	MO	55	98	76

**Basic combinations**	MO-or-iEDWT	73	97	85
	
	T2 hyperintensity or (MO-or-iEDWT)	94	66	80
	
	T2 hyperintensity and (MO-or-iEDWT)	69	100	84

**New algorithm**		92	90	91

**Figure 1:**
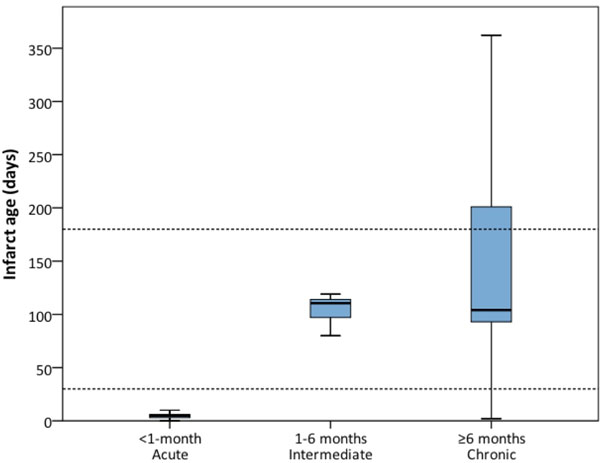
Range infarct age for categories based on the new algorithm

An additional benefit of the algorithm was the ability to identify intermediate-aged-MI (1-6-month-old). This was based on finding T2-hyperintensity-size < DE-infarct-size, and when present, patients had median infarct age of 110 days (IQR: 96, 115) (Figure 1).

## Conclusions

A novel algorithm incorporating components of a routine CMR scan improves the determination of infarct age compared with T2-CMR alone. Certain CMR findings may be specific for intermediate-aged MI.

## Funding

None.
